# PW01-035 – Mutations in PB30.2D and complexing with caspase-1

**DOI:** 10.1186/1546-0096-11-S1-A88

**Published:** 2013-11-08

**Authors:** K Nazaryan, G Arakelov

**Affiliations:** 1 Russian-Armenian (Slavonic) University, Yerevan, Armenia; 2 Institute of Molecular Biology, Yerevan, Armenia

## Introduction

Mutations M680I, M694V and V726A of Pyrin - the product of MEFV gene - are localized at the domain B30.2 (PB30.2D) and responsible for manifestation of the most widespread and severe forms of FMF. From the other hand, it is well known that malfunction of the pyrin-caspase-1complex is the main reason for inflammation during FMF. Therefore, we suggest that comparative investigation of normal and mutated pyrin and caspase-1 interaction will help to reveal possible link between those mutations and structural changes that influence formation of pyrin-caspase-1 complex.

## Objectives

From abovementioned the goal of current study was to detect possible changes in the tertiary structure of B30.2 and to show structural consequences that influence formation of pyrin-caspase-1 complex.

## Methods

3D structures of M680I, M694V and V726A was build up with the help of Rosetta software, caspase-1and B30.2 files obtained from PDB. Alignment of native B30.2 with three mutant forms has been done by VMD programme. In silico molecular modeling interaction experiments between native and mutated B30.2 and caspase-1 has been performed by CHARMM software in in Effective Energy Function 1 environment using 24-node computer cluster. Each molecular modeling time was 110ns with iteration 2fs.

## Results

Influence of mutations on B30.2 tertiary structure has revealed the following structural changes: M680I – induces conversion of β-sheet into loop in position THR663-TRP665, loop - β-sheet in THR707- LEU710 and loop - α-helix in LYS765 – ALA768 site. Alignment of the native structure with mutation has RMSD=1,137 Å. M694V – conversion of the loop into β-sheet in the sites LYS695-GLU696 and THR707 – LEU709, RMSD=1,699 Å. V726A – conversion β-sheet into loop in the sites TRP655 – VAL657, loop - β-sheet in THR707- LEU710 and ARG725 – GLY727, loop - α-helix in ASP762 – LYS765, RMSD=1,808 Å. Molecular modeling of B30.2-caspase-1 dynamic interaction has shown significant differences between interaction energy of normal and mutated domains. Decrease in the minimal and average complex formation energy in the case of V726A (71 and 69% in comparison to the normal domain), no changes for M680I (99 and 97.4%) and increase for M694V (136.5 and 126,5%). At the same time maximal energy values have not shown any considerable differences.

## Conclusion

Summarizing results of impact of the mutations on the B30.2-caspase-1 complex formation we came to the conclusion that dramatic changes in the tertiary structures which reflected in the shifts of binding sites and differences of interaction energy have notable influence on the complex formation, which in its turn should affect process of IL1β activation a trigger stage of inflammation process.

## Disclosure of interest

None declared

